# Editorial: Hematopoietic stem cell transplantation: back to the future

**DOI:** 10.3389/fmed.2024.1390041

**Published:** 2024-03-18

**Authors:** Erden Atilla

**Affiliations:** Translational Science and Therapeutics Division, Fred Hutchinson Cancer Center, Seattle, WA, United States

**Keywords:** hematopoietic stem cell transplantation, acute graft vs. host disease, gastrointestinal GVHD, GVHD prophylaxis, hypomethylating agents, XMEN syndrome

“In the 1960s in particular and even into the 1970s, there were very responsible physicians who said this would never work,” E. Donnall Thomas, the father of bone marrow transplantation, mentioned in an interview after he was awarded with the Nobel Prize for the discovery and development of hematopoietic stem cell transplantation (HSCT). After more than 60 years and more than 1.5 million procedures, HSCT is the only example of a cell immunotherapy procedure used on a wide scale worldwide (1). Some of the tremendous efforts to improve survival in HSCT include attempts to refine donor selection, manage graft vs. host disease (GVHD) treatment and prevention, choose potent anti-fungal and anti-viral strategies and modulate the intensity of the conditioning regimen (2). Here, we discuss recent advances and novel approaches in the field of HSCT.

Shahzad et al. conducted the first bibliometric analysis to demonstrate the 100 most cited clinical papers on HSCT. This article highlighted the current state of the art and identified directions for future clinical research. Among the top 100, only three articles were cited more than 2,000 times, and the research was mostly from North American and European institutions. Their findings showed most highly cited papers were not cited until 1968, although the first human bone marrow transfusion was performed in 1939 (3). There was a peak in publications between 1991 and 2000.

Acute graft vs. host disease (aGVHD), a major complication of HSCT, represents a significant cause of morbidity and mortality following allogeneic HSCT (4). Di Francesco et al. compared aGVHD and its outcome in three consecutive time frames. This valuable study showed how bone marrow transplantation trends have advanced throughout the years. The breakthrough in this study was the reduction of transplant-related mortality by improvements in prevention and treatment of GVHD. Significant predictive factors found for grade II-IV GVHD were anti-thymocyte globulin (ATG), post-transplant cyclophosphamide, a family mismatched donor, a matched unrelated donor, an unrelated mismatched donor, a donor above 40 years, and hematological malignancy rather than aplastic anemia. Gao et al. reported a single-center prospective experience of GVHD prophylaxis for patients undergoing haploidentical allogeneic HSCT combining post-transplantation cyclophosphamide (PTCy) and tacrolimus as well as low-dose post-engraftment anti-thymoglobulin (ATG). This work was based on their previous phase II study using PTCy-cyclosporine (CsA) undergoing allogeneic HSCT with peripheral blood stem cell and myeloablative conditioning. Since the incidence of acute GVHD remained high in a haploidentical setting, they replaced CsA with tacrolimus and added a single low dose of ATG after neutrophil engraftment (5). They observed a low incidence of grade II-IV aGVHD, no cases of grade III-IV aGVHD at day 100 and no cases of steroid-resistant aGVHD. However, a relatively high incidence of CMV reactivation was noted due to immunosuppression. After the skin, the gastrointestinal tract is the second-most affected organ in aGVHD (6). In another article, Kim et al. investigated the outcomes in 51 pediatric patients following allogeneic HSCT according to the clinical, endoscopic, and histologic severity of their gastrointestinal GVHD. A Cox proportional-hazards regression analysis showed that the groups with more severe clinical and histologic gastrointestinal GVHD had a higher risk of non-relapse mortality and lower 5-year overall survival rates.

XMEN disease is a rare entity characterized by immune dysregulation marked by autoimmune conditions, infections and EBV-associated lymphoproliferative disorders (7). de Groot et al. described a case with XMEN syndrome following autologous HSCT due to mutations in the *MAGT1* gene presenting with immune thrombocytopenia, EBV-associated malignancy, recurrent airway infections, and bronchiectasis. The authors underlined the pre-emptive application of allogeneic HSCT for curative treatment.

To prolong disease-free survival following allogeneic HSCT in acute myeloid leukemia and myelodysplastic syndrome, hypomethylating agents (HMAs) are suitable for post allogeneic HSCT maintenance. HMAs mediate a direct anti-leukemic effect, induce a CD8+ tumor-specific T cell response, expand regulatory T cells and enhance the graft vs. leukemia effect epigenetically (8). To evaluate HMAs for post-allogeneic HSCT maintenance, Kungwankiattichai et al. performed a meta-analysis. In the HMA maintenance group, the overall survival and the relapse-free survival were superior; the cumulative incidence of relapse and non-relapse mortality were lower than in the observation group. However, some limitations of this study were reported in a commentary by Beauvais et al. (9), who believed an inaccurate approach to data design and analyses led to an immortal time bias. These authors argued that only a randomized study can demonstrate a definitive conclusion.

In summary, HSCTs have been accepted as standard care for various hematologic malignancies. Ongoing research has focused on strategies to improve response rates while minimizing complications.

## Author contributions

EA: Writing—original draft, Writing—review & editing.
